# FHIT down-regulation was inversely linked to aggressive behaviors and adverse prognosis of gastric cancer: a meta- and bioinformatics analysis

**DOI:** 10.18632/oncotarget.22369

**Published:** 2017-11-03

**Authors:** Hua-Chuan Zheng, Li-Li Liu

**Affiliations:** ^1^ Department of Experimental Oncology and Animal Center, Shengjing Hospital of China Medical University, Shenyang 110004, China; ^2^ Department of Pathology, Harbin Medical University-Daqing, Daqing 163319, China

**Keywords:** FHIT, gastric cancer, meta-analysis, bioinformatics analysis

## Abstract

FHIT (fragile histine triad) acts as diadenosine P1, P3-bis (5'-adenosyl)-triphosphate adenylohydrolase involved in purine metabolism, and induces apoptosis as a tumor suppressor. We performed a systematic meta- and bioinformatics analysis through multiple online databases up to March 14, 2017. The down-regulated FHIT expression was found in gastric cancer, compared with normal mucosa and dysplasia (*p* < 0.05). FHIT expression was negatively with depth of invasion, lymph node metastasis, distant metastasis, TNM staging and dedifferentiation of gastric cancer (*p* < 0.05). A positive association between FHIT expression and favorable overall survival was found in patients with gastric cancer (*p* < 0.05). According to Kaplan-Meier plotter, we found that a higher FHIT expression was negatively correlated with overall and progression-free survival rates of all cancer patients, even stratified by aggressive parameters (*p* < 0.05). These findings indicated that FHIT expression might be employed as a potential marker to indicate gastric carcinogenesis and subsequent progression, even prognosis.

## INTRODUCTION

*FHIT* (fragile histine triad) is also known as human accelerated region 10 and encompasses the common fragile site FRA3B at 3p14.2, where carcinogen-induced damage can lead to translocations and aberrant transcripts of this gene. It spans more than 1.5 mb as the second largest human gene, and encodes a 17kd diadenosine P1, P3-bis (5'-adenosyl)- triphosphate adenylohydrolase involved in purine metabolism. Moreover, FHIT protein inhibits cell proliferation and induces apoptosis as a tumor suppressor, which is independent of its hydrolase activity [1]. FHIT up-regulates the expression of thymidine kinase 1, resulting in dNTP imbalance, and spontaneous replication stress that leads to chromosomal aberrations, allele copy number variations, insertions/deletions, and single-base substitutions [2]. FHIT acts as a checkpoint in cell proliferation mediated by activated tyrosine kinase receptors that recruit src [3]. Src family tyrosine kinases can phosphorylate FHIT at tyrosine 114 (Y114) within the unstructured loop C-terminal of the catalytic site, which induces Caspase- dependent apoptosis of lung cancer cells by decreasing survivin expression and Akt activity [4]. FHIT also associates with the lymphoid enhancer-binding factor 1/T cell factor/ β-catenin complex by directly binding to β-catenin, and repressing the transcription of cyclin D1, axin2, MMP-14, and survivin [5]. FHIT delocalizes Annexin a4 from plasma membrane to cytosol and sensitizes lung cancer cells to paclitaxel [6]. Semba et al. [7] found that FHIT overexpression down-regulated cyclophilin A expression to prevent cyclophilin A-induced up-regulation of cyclin D1, Cdk4, resulting in G_1_-S progression. *FHIT* gene expression is repressed in breast cancer cells by mitogenic signaling through PI3K/Akt/FOXO pathway [8], which promots MHC-I down-regulation and allows escape from immunosurveillance [9] and enhances expression of oxidative stress response genes after exposure to cigarette smoke extract, including cytoprotective enzyme heme oxygenase 1 [10].

The transcription deletion of *FHIT* was detectable in intrahepatic cholangiocellular carcinomas induced by N-nitrosobis (2-oxopropyl) amine in female Syrian golden hamsters [11]. *FHIT*-deficient mice easily developed forestomach tumors and invasive bladder carcinoma after the treatment with N-nitrosomethylbenzylamine and N-butyl-N- (4-hydroxybutyl) nitrosamine respectively [12, 13]. BBN(N-butyl-N-(-4-hydroxybutyl)- nitrosamine)-induced urinary bladder cancer could be prevented in *FHIT* knock-out mouse by Rofecoxib, a cox-2 inhibitor [14]. Lung cancer susceptibility in *FHIT*-deficient mice was increased by Vhl haploinsufficiency [15]. Fujishita et al. [16] found that *FHIT* knockout mice developed tumors in the lymphoid tissue, liver, uterus, testis, forestomach and small intestine. In the present study, we performed a meta- and bioinformatics analysis to clarify the clinicopathological and prognostic significances of FHIT expression in gastric cancer at both mRNA and protein levels.

## RESULTS

### Characteristics of eligible studies

**Figure 1:**
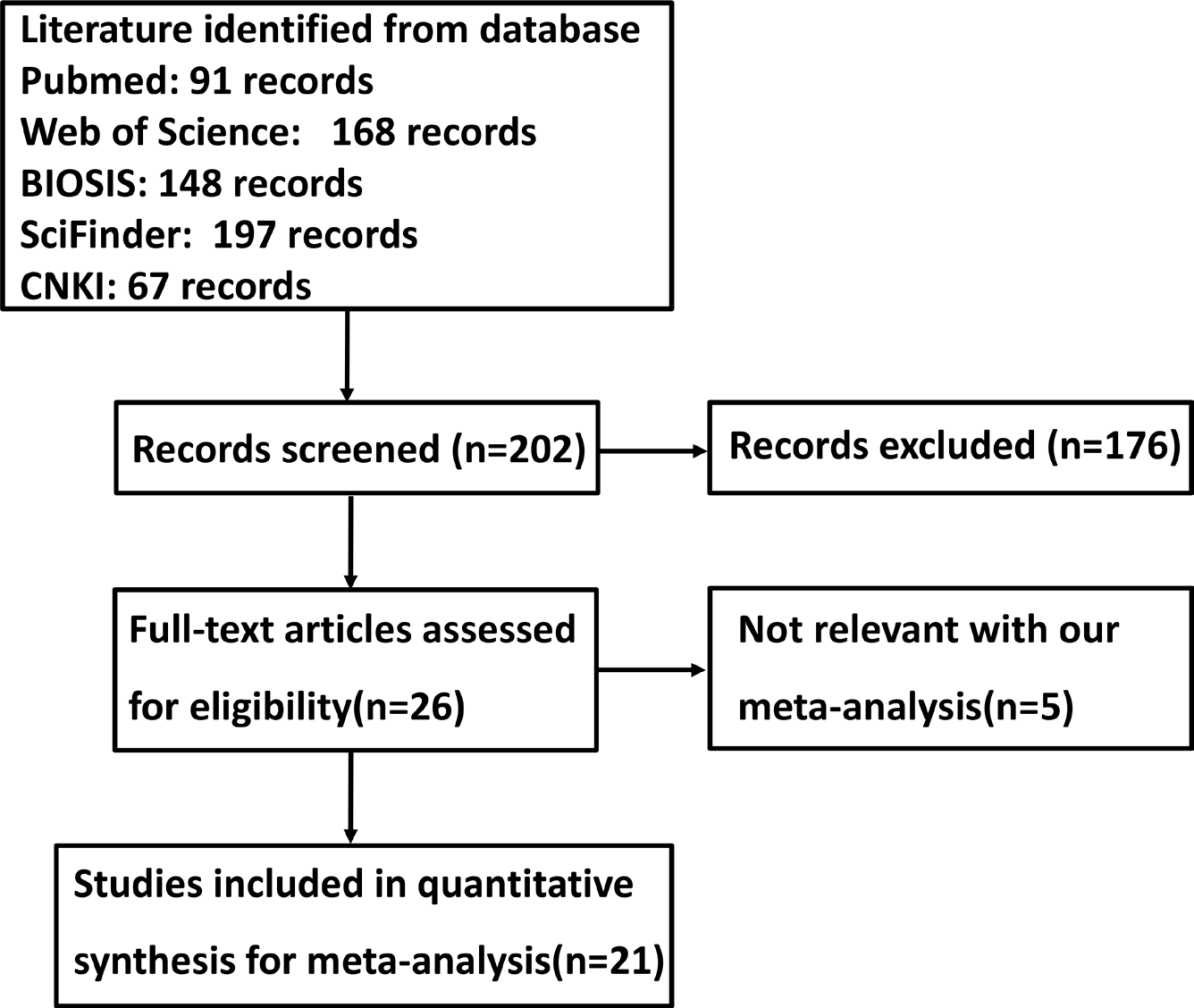
Flow diagram of the selection process in this meta-analysis

Figure 1 is a flow diagram of article selection for our meta-analysis. As shown in Table 1, a total of 21 articles on the relationship between FHIT expression and cancer risk, clinicopathological or prognostic parameters of gastric cancer were retrieved for our meta-analysis by immunohistochemistry from PubMed, Web of Science, BIOSIS, SciFinder and CNKI. Only 10 articles contained the samples of normal gastric mucosa [17–26] and 7 did gastric precancerous lesion-dysplasia [17, 22, 25–29]. There appeared the comparison between FHIT expression and clinicopathological characteristics of gastric cancer in 19 pieces of paper, including sex, depth of invasion, lymph node metastasis, distant metastasis, TNM staging and Lauren's classification [17–36]. Finally, we discussed the prognostic significance of FHIT expression in 7 articles [20, 25, 30–32, 36, 37].

**Table 1:** Main characteristics of eligible studies

First author	Year	Country	Ethnicity	Antibody Source	Cases	Control	Risk to cancer	Outcome	Quality
Bria E	2013	Italy	European	Neomarker	208	–	–	Positive	8
Zheng HC	2010	Japan	Asian	Neomarker	377	–	–	–	8
Czyzewska J	2009	Poland	European	Abcam	80	–	–	–	8
Zheng HC	2008	Japan	Asian	Neomarker	270	–	–	–	8
Zheng HC	2007	Japan	Asian	Neomarker	242	49	Down	Positive	9
Hara A	2007	Japan	Asian	IBL	103	–	–	–	8
Bragantini E	2006	Italy	European	Zymed	362	–	–	Positive	8
Zhao P	2005	China	Asian	Zymed	76	76	Down	Positive	9
Kawaguchi K	2004	Japan	Asian	IBL	55	–	–	–	8
Skopelitou AS	2003	Greece	European	Zymed	59	–	–	–	8
Lee HS	2003	Korea	Asian	Zymed	329	–	–	Positive	8
Rocco A	2003	Germany	European	Zymed	137	–	–	–	8
Capuzzi D	2000	USA	American	Self-making	55	–	–	Positive	8
Yang Y	2016	China	Asian	Zhongshan	86	33	Down	–	9
Chang TM	2013	China	Asian	Zhongshan	30	60	Down	–	8
Ma YY	2011	China	Asian	Zhongshan	70	30	Down	–	8
Li DX	2009	China	Asian	Zhongshan	50	30	Down	–	8
Zhu SZ	2009	China	Asian	Zhongshan	52	48	Down	–	8
Li YZ	2009	China	Asian	Zymed	336	60	Down	Positive	9
Guo XQ	2006	China	Asian	Santa Cruz	80	80	Down	–	8
Wang P	2002	China	Asian	Zhongshan	78	–	Down	–	8

Down, down-regulated expression.

### Association between FHIT expression and cancer susceptibility of gastric mucosa or dysplasia

**Figure 2:**
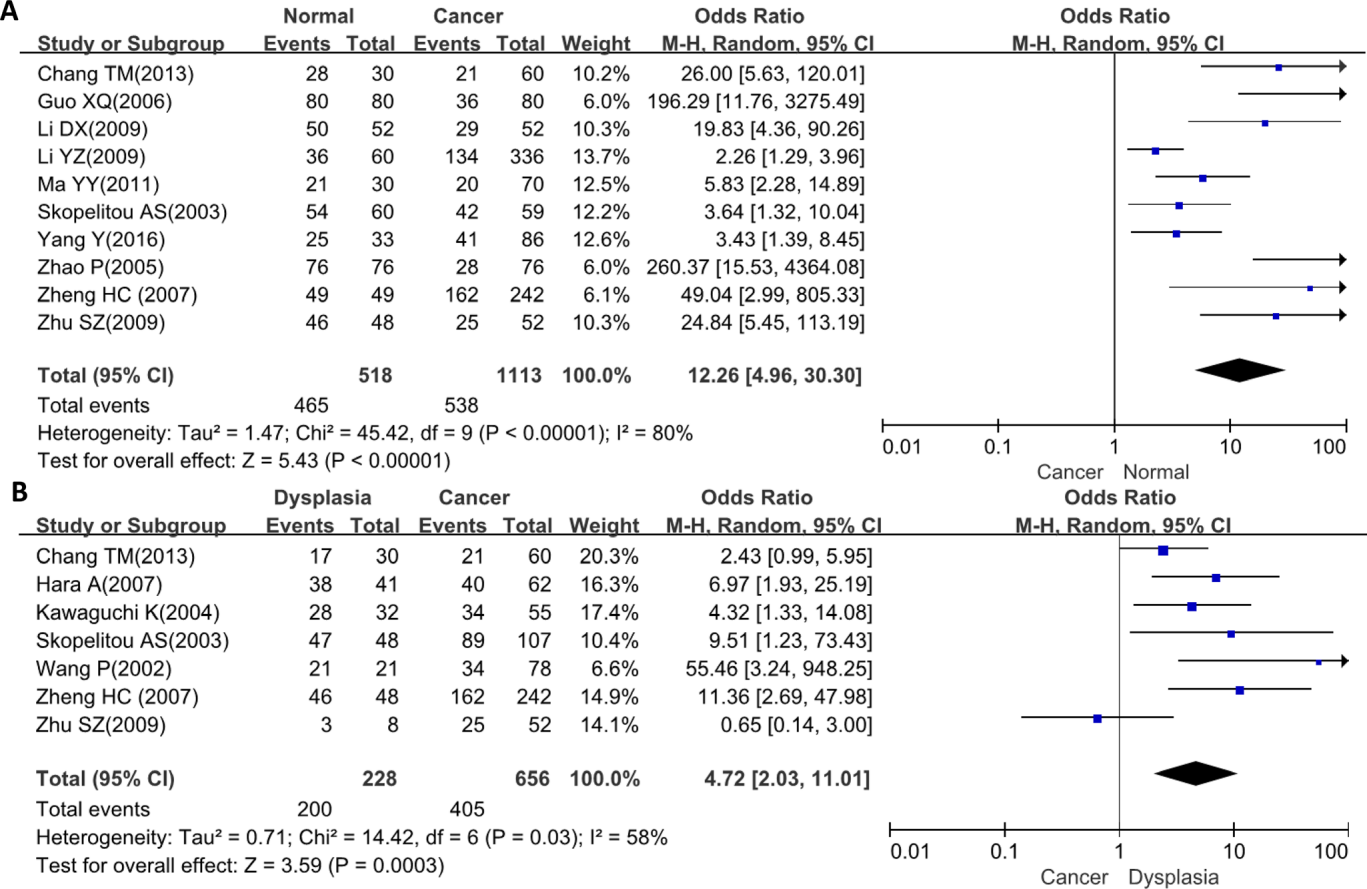

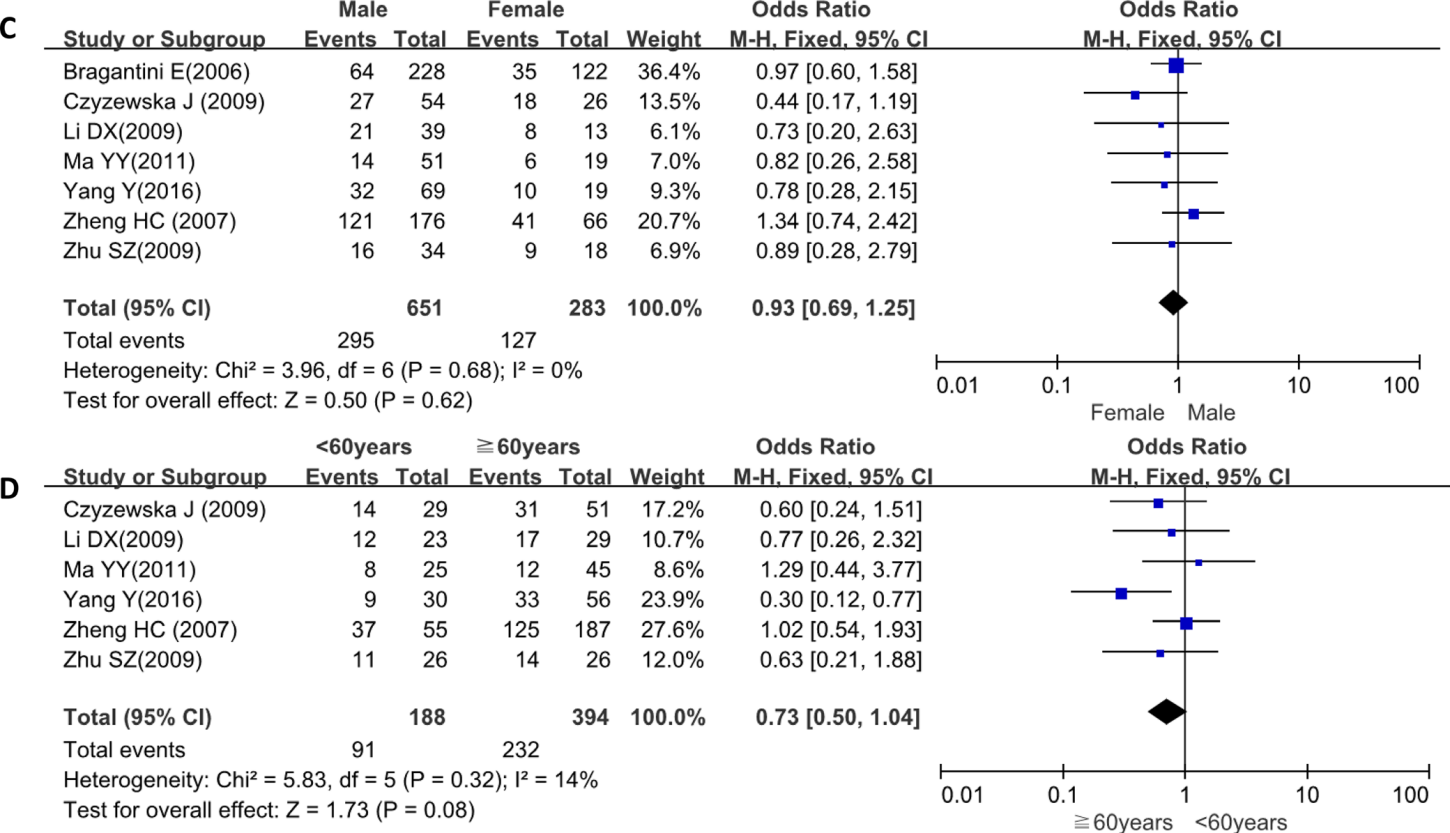

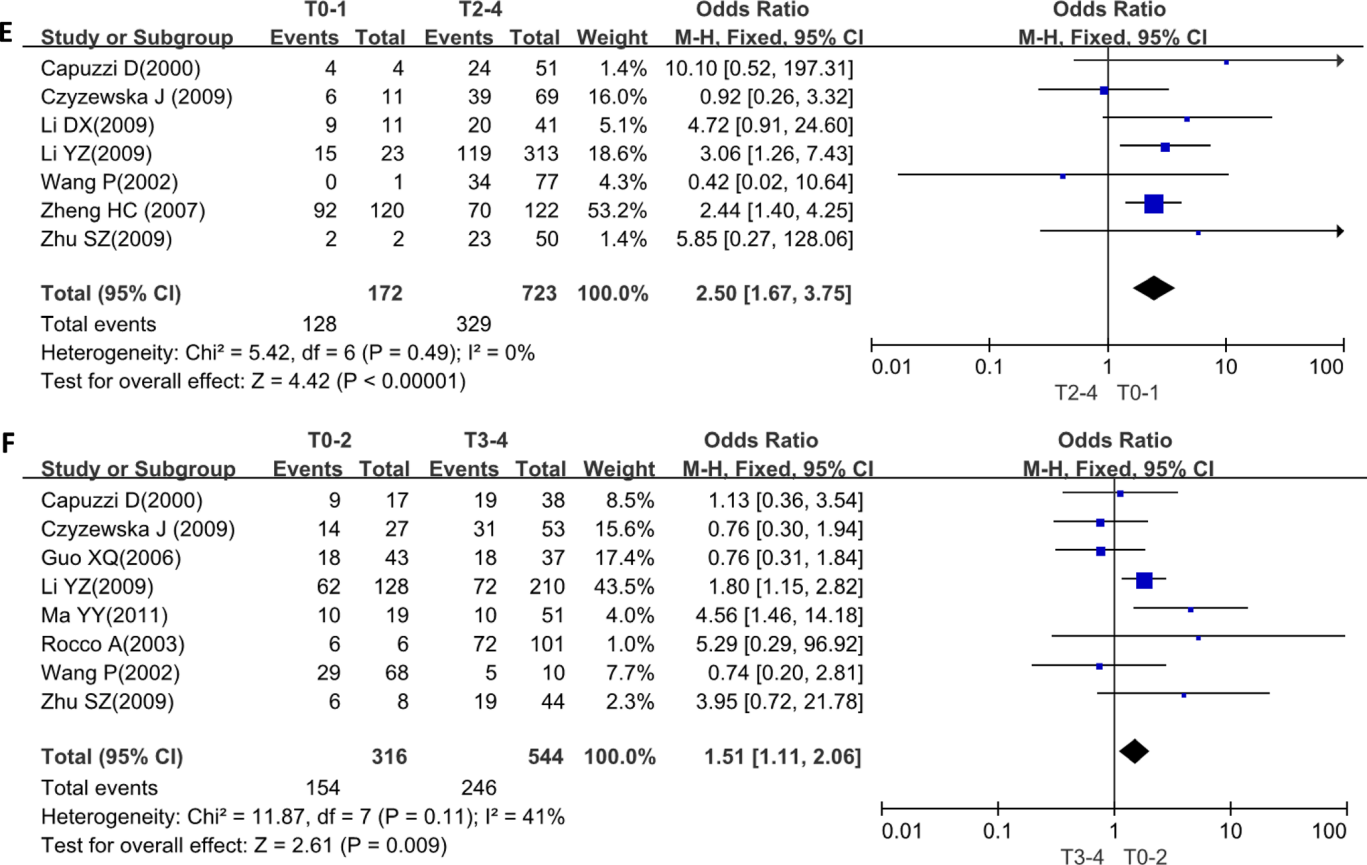

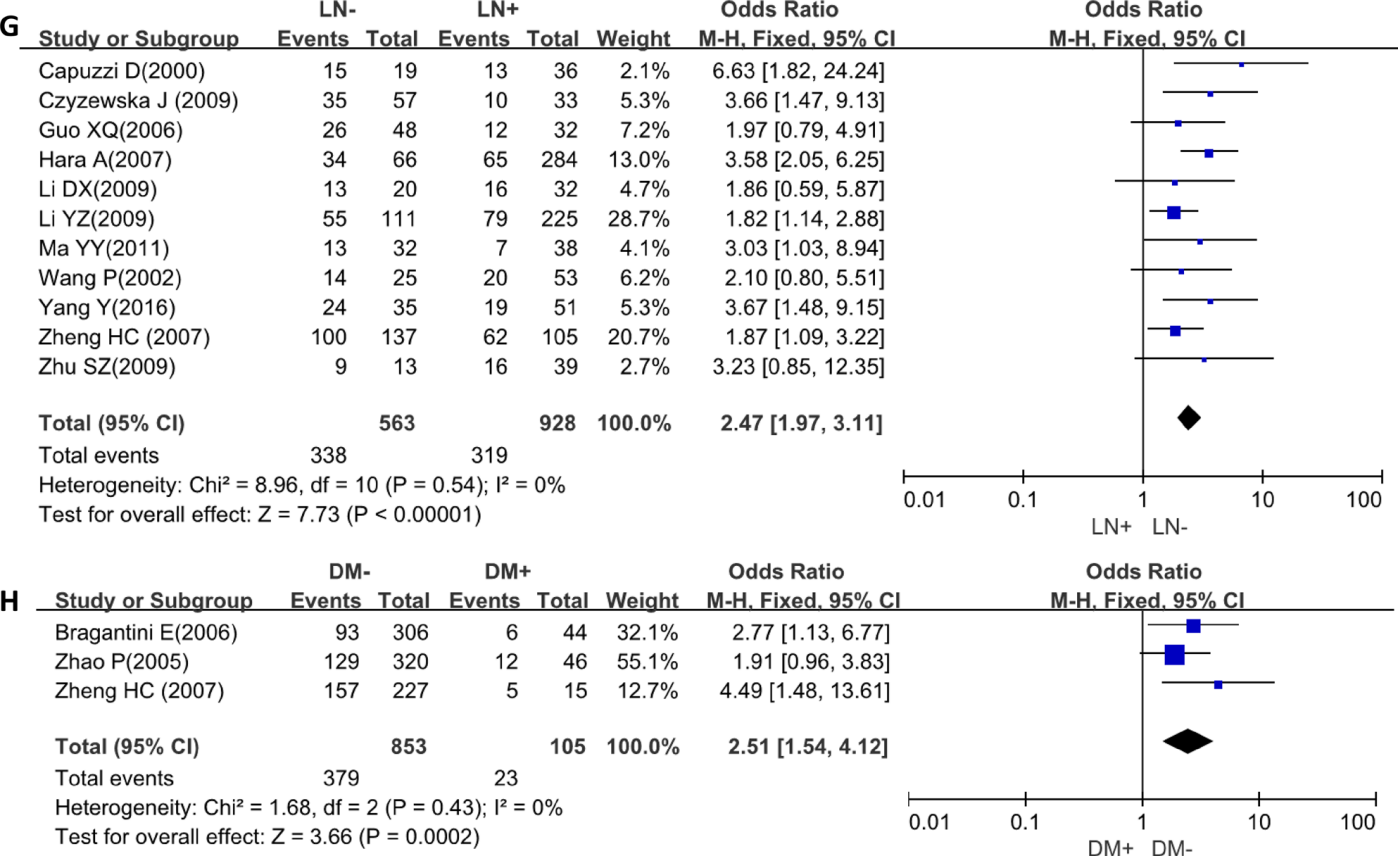

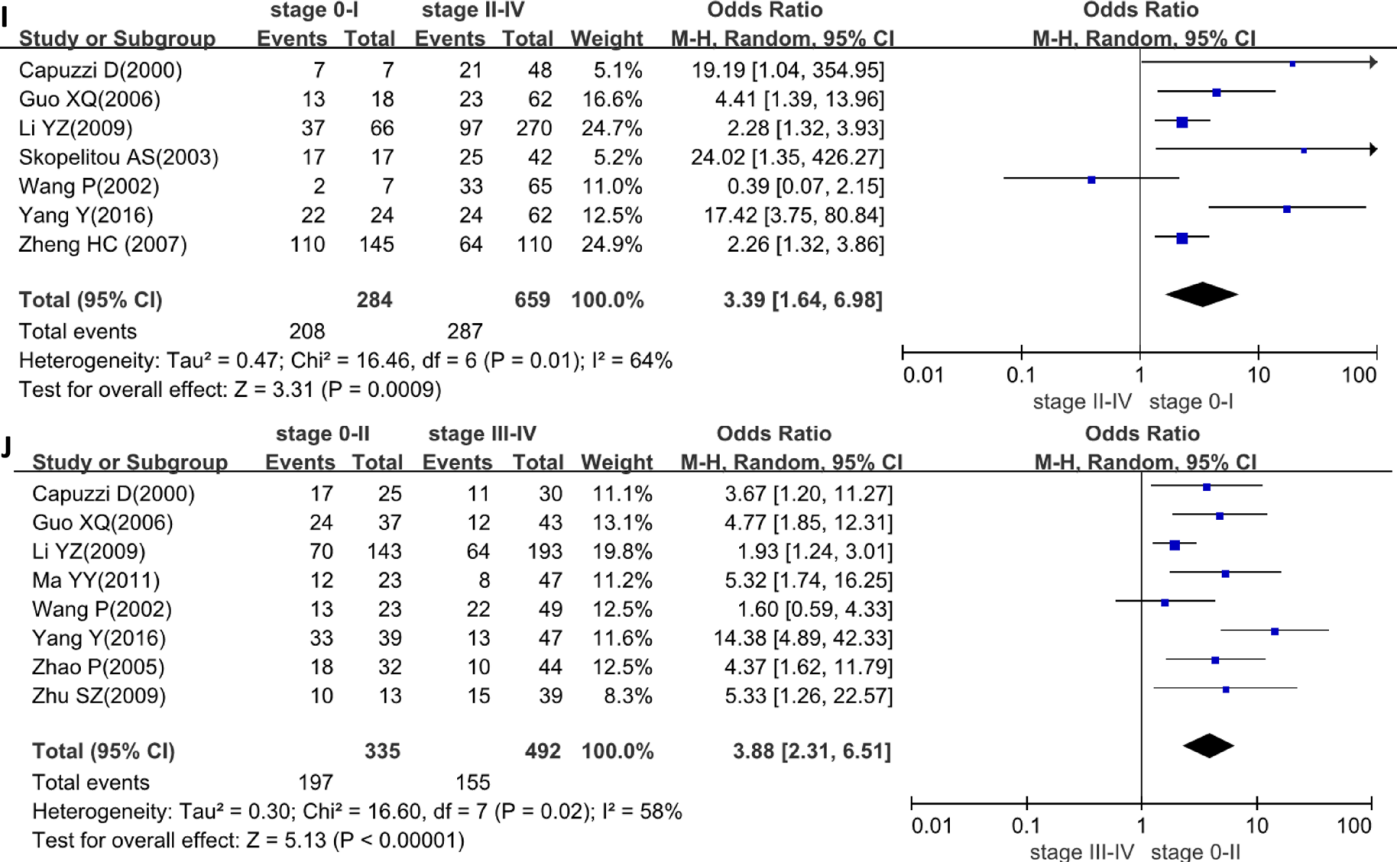

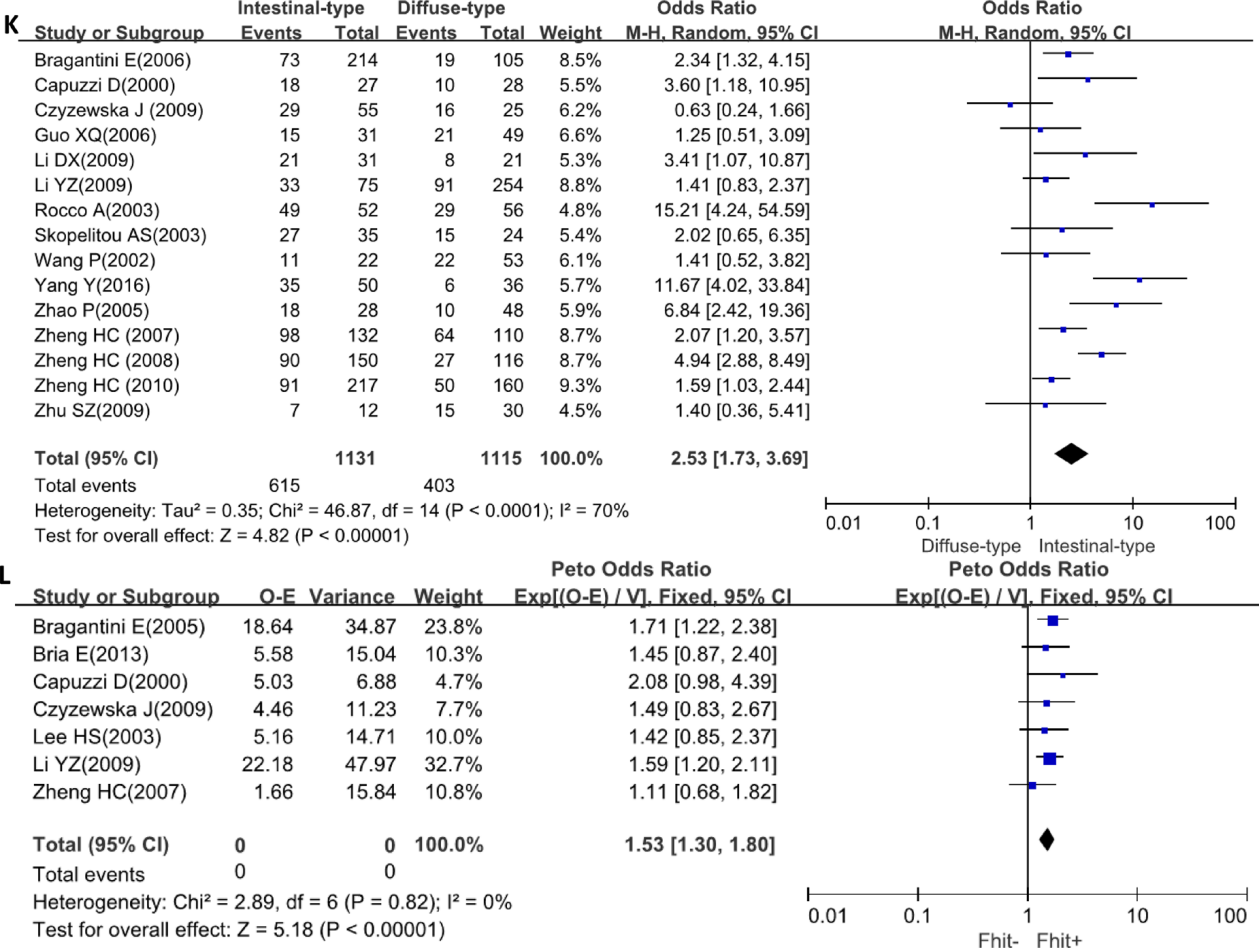
Forest plot for the relationship between FHIT expression and clinicopathological parameters of gastric cancer (**A**), Gastric carcinogenesis (cancer vs normal mucosa); (**B**), gastric carcinogenesis (cancer vs dysplasia); (**C**), correlation between sex and FHIT expression (male vs female); (**D**), correlation between age and FHIT expression (< 60years vs ≧ 60years); (**E**), correlation between depth of invasion and FHIT expression (T0-1 vs T2-4); (**F**), correlation between depth of invasion and FHIT expression (T0-2 vs T3-4); (**G**), correlation between lymph node metastasis (LN) and FHIT expression (LN- vs LN+); (**H**), correlation between distant metastasis (DM) and FHIT expression (DM- vs DM+); (**I**), correlation between TNM staging and FHIT expression (stage 0-I vs II-IV); (**J**), correlation between TNM staging and FHIT expression (stage 0-II vs III-IV); (**K**), correlation between differentiation and FHIT (intestinal-type vs diffuse-type); (**L**), correlation between survival and FHIT expression (FHIT- vs FHIT+).

We analyzed the association between FHIT expression and cancer susceptibility of gastric normal mucosa in 10 studies with 1113 cancers and 518 controls. As a result, we found down-regulated FHIT expression in gastric cancer, compared with normal mucosa (Figure 2A, *p* < 0.00001). Additionally, the cancer risk of FHIT-negative dysplasia was also analyzed and the same trend was observed as gastric mucosa using 656 cancer and 228 dysplasia (Figure 2B, *p* = 0.0003).

### Association between FHIT expression and clinicopathological parameters of gastric cancer

There was no difference in FHIT expression between male and female patients with gastric cancer (Figure 2C, *p* > 0.05) or between younger and elder cancer patients (Figure 2D, *p* > 0.05). A higher FHIT expression was detected in T0-1 than T2-4 gastric cancer (Figure 2E, *p* < 0.00001) or T0-2 than T3-4 ones (Figure 2F, *p* = 0.009). FHIT expression was negatively related to lymph node metastasis (Figure 2G, *p* < 0.00001) and distant metastasis (Figure 2H, *p* = 0.0002) of gastric cancer. Stage 0–I and 0–II cancers showed higher FHIT expression than stage II–IV (Figure 2I, *p* = 0.0009) and III-IV (Figure 2J, p < 0.00001) respectively. FHIT protein was more expressed in intestinal-type than diffuse-type carcinomas (Figure 2K, *p* < 0.00001).

### Association between FHIT expression and survival rate of gastric cancer

As indicated in Figure 2L, the pooled result from 7 datasets demonstrated a positive association between FHIT expression and favorable overall survival in the patients with gastric cancer (HR = 1.53, 95% CI: 1.30–1.80, *p* < 0.0001).

### Publication bias

**Figure 3:**
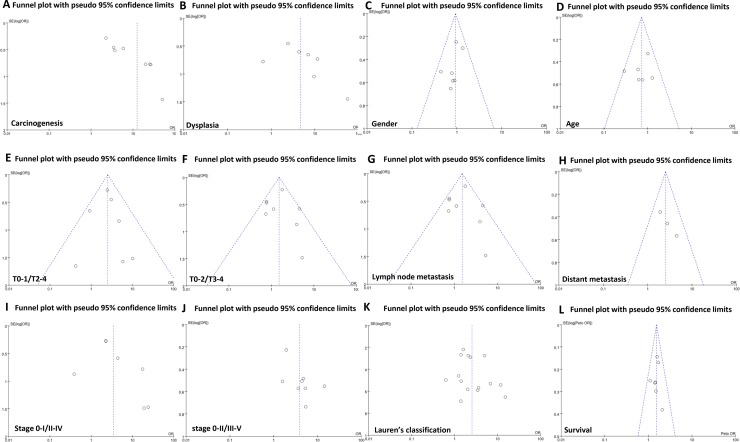
Funnel plot for publication bias test between FHIT expression and gastric carcinogenesis or progression The bias was analyzed about risk degrees of FHIT expression in gastric mucosa (**A**) and dysplasia (**B**) for gastric carcinogenesis. Additionally, it was tested between FHIT expression and clinicopathological features of gastric cancer, including gender (**C**), age (**D**), depth of invasion (**E** and **F**), lymph node metastasis (**G**), distant metastasis (**H**), TNM staging (**I** and **J**), differentiation (**K**) and prognosis (**L**).

The heterogeneity test was performed as shown in Figure 3. Sensitivity analysis was used to evaluate individual study's influence on the pooled results by deleting one single study each time from pooled analysis. As a result, the correlation between FHIT expression and TNM staging in Wang's study had a significant effect on the pooled OR as shown in Figure 2I. When this study was excluded, the heterogeneity test was significantly reduced (data not shown).

### The clinicopathological and prognostic significances of FHIT mRNA expression in gastric cancers

**Figure 4:**
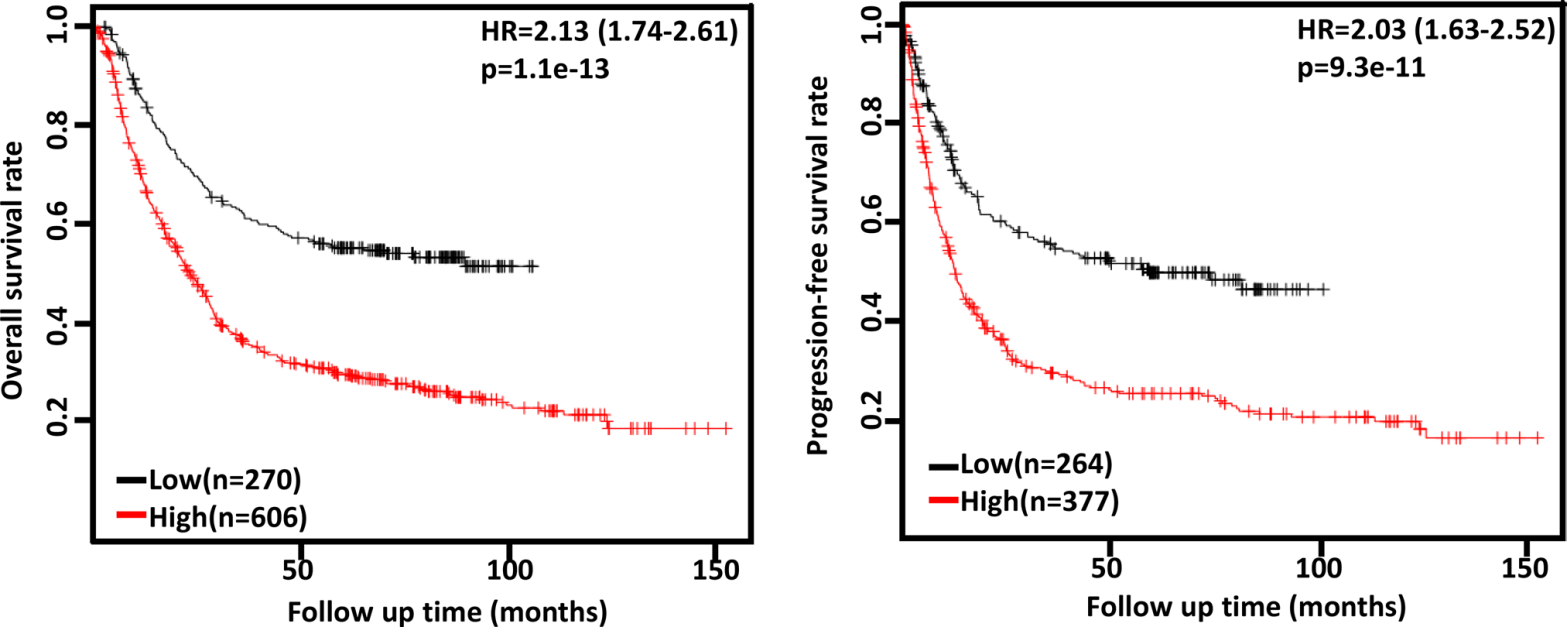
The prognostic significance of *FHIT* mRNA expression in gastric cancer According to the data from Kaplan-Meier plotter, *FHIT* mRNA expression was negatively related to both overall and progression-free survival rates of the patients with gastric cancer (*p* < 0.05). HR, hazard ratio.

According to Oncomine dataset, there was no difference in FHIT mRNA expression between gastric mucosa and cancer (*p* > 0.05, data not shown). In TCGA data, FHIT mRNA expression was not correlated with any clinicopathological parameter of gastric cancer (*p* > 0.05, data not shown). According to Kaplan-Meier plotter, we found that a higher FHIT mRNA expression was negatively correlated with overall and progression-free survival rates of all cancer patients, even stratified by gender and Her2 status (Figure 4 and Table 2, *p* < 0.05). It was the same for the N1-3, N1, stage II and III, diffuse-type cancer patients (Table 2, *p* < 0.05). M0 and intestinal-type cancer patients with high FHIT mRNA expression showed a short overall survival time than those with its low expression (Table 2, *p* < 0.05). There appeared a negative relationship between FHIT mRNA expression and the progression-free survival rate of T4 cancer patients (Table 2, *p* < 0.05).

**Table 2:** The prognostic significance of *FHIT* mRNA in gastric cancer

Clinicopathological features	Overall survival		Progression-free survival	
	Hazard ratio	*p*	Hazard ratio	*p*
Sex				
Female	1.7 (1.17−2.45)	0.0044	1.62 (1.1−2.38)	0.013
Male	1.61 (1.3−2.01)	1.7e−05	1.52 (1.17−1.96)	0.0016
T				
2	1.3 (0.83−2.01)	0.25	1.19 (0.76 − 1.85)	0.44
3	1.33 (0.94−1.89)	0.11	1.17 (0.82−1.66)	0.39
4	3.38 (1.29−8.88)	0.0096	5.09 (1.65−15.65)	0.0021
N				
0	1.76 (0.75−4.17)	0.19	1.69 (0.71−3.98)	0.23
1–3	1.46 (1.1−1.94)	0.0079	1.37 (1.04−1.8)	0.024
1	2.17 (1.37−3.44)	7e−04	1.79 (1.21−2.65)	0.0032
2	1.55 (0.92−2.64)	0.099	1.39 (0.87−2.2)	0.17
3	0.73 (0.41−1.28)	0.27	0.67 (0.38−1.17)	0.15
M				
0	1.43 (1.08−1.91)	0.013	1.26 (0.97−1.65)	0.083
1	1.69 (0.89−3.21)	0.11	1.38 (0.71−2.67)	0.34
TNM staging				
I	2.81 (0.96−8.26)	0.051	1.98 (0.62−6.28)	0.24
II	1.94 (1.06−3.53)	0.028	0.5 (0.24−1.01)	0.049
III	1.81 (1.35−2.43)	5.3e−05	1.84 (1.24−2.72)	0.0021
IV	0.71 (0.46−1.08)	0.11	0.64 (0.42−0.97)	0.034
Lauren's classification				
Intestinal-type	1.74 (1.27−2.39)	0.00053	0.81 (0.55−1.2)	0.3
Diffuse-type	1.65 (1.17−2.33)	0.0038	1.52 (1.07−2.14)	0.017
Mixed-type	2.41 (0.87−6.68)	0.082	1.71 (0.55−5.39)	0.35
Her2 positivity				
-	1.62 (1.29−2.02)	2.5e−05	1.42 (1.08−1.87)	0.01
+	1.37 (1.03−1.83)	0.031	1.47 (1.05−2.05)	0.023
Treatment				
Surgery alone	1.27 (0.94−1.7)	0.11	1.15 (0.85−1.56)	0.37
5-FU-based adjuvant	0.73 (0.51−1.03)	0.071	0.7 (0.5−0.99)	0.044
Other adjuvant	0.44 (0.17−1.1)	0.07	0.48 (0.22−1.07)	0.068

## DISCUSSION

FHIT can interact with Gαq to enhance the growth inhibitory effect of FHIT [38], and with ubiquitin-conjugating enzyme 9 to inhibit its diadenosine triphosphate hydrolase activity [39, 40]. FHIT induces autophagy and apoptosis in lung cancer cells, and suppresses the growth epithelial-mesenchymal transition (EMT) and metastasis [41–43]. Further investigation showed that FHIT inhibited EMT through an EGFR/Src/ERK/Slug signaling axis in human bronchial cells [44]. Huang et al. [45] found that FHIT suppressed proliferation and promoted apoptosis in cholangiocarcinoma cells by blocking PI3K-Akt pathway. Nakagawa et al. [46] demonstrated that FHIT overexpression suppressed colorectal cell viability, and resulted in a higher sensitivity to oxidative stress evoked by inhibitors of mitochondrial electron transport. Vecchione et al. [13] found FHIT overexpression inhibited cell growth, increased apoptotic cell population, and suppressed tumor growth in nude mice of the bladder cancer cells. To investigate the clinicopathological and prognostic significances of FHIT expression, we analyzed 21 studies, which met specific inclusion criteria and had moderate to high quality according to their NOS scores.

Consistent with the data about colorectal cancer [47], bladder cancer [48], thyroid cancer [49] and lung cancer [50], we found down-regulated FHIT expression in gastric cancer, compared with gastric mucosa or dysplasia in the present study, suggesting that FHIT hypoexpression contributes to gastric carcinogenesis as a late event. Huang et al. [51] found that cervical microinvasive and invasive carcinomas had significantly lower FHIT expression than normal epithelium and dysplasia. Kuwai et al. [52] demonstrated that FHIT protein expression were lower in invasive colorectal carcinoma than in adenoma and carcinoma *in situ*. Combined with these findings, it was suggested that gradual FHIT down-regulation promoted gastric carcinogenesis. Reportedly, *FHIT* down-regulation or loss might be due to its promoter methylation and loss of heterozygosity in cancers, but not its gene mutation [47, 49, 53, 54].

Here, our meta-analysis showed FHIT expression was inversely linked to depth of invasion, lymph node metastasis, distant metastasis and TNM staging of gastric cancer, indicating that its hypoexpression promoted invasion and metastasis of gastric cancer, in agreement with the reports about oral squamous cell carcinoma [55], cholangiocarcinoma [56], colorectal cancer [57], and cervical cancer [58]. *FHIT* promoter hypermethylation was positively linked to tumor staging, pathological grade, or lymph node metastasis of colorectal cancer [59], thyroid cancer [49] and lung cancer [60]. Chen et al. [61] found that *FHIT* mRNA expression had an inverse relation with larger invasive range, poor histological differentiation and advanced clinical stage of nasopharyngeal carcinoma. Yin et al. [54] reported that homozygous deletion of exon 5 of *FHIT* was positively associated with lymph node metastasis of differentiated thyroid cancer and the homozygous deletion of exon 8 was positively associated with the tumor pathological grade, TNM staging, and lymph node metastasis of differentiated thyroid cancer. Taken together, we concluded that FHIT loss might be employed as a potential biomarker for aggressiveness of gastric cancer.

Reportedly, FHIT expression was positively related to the favorable prognosis of the patients with lung cancer [62], oral squamous cell carcinoma [55], head and neck squamous cell carcinoma [63], diffuse large B-cell lymphoma [64] and cervical cancer [51]. It might be also demonstrated to indicate the favorable prognosis of cholangiocarcinoma [58], non-small cell lung cancer [65], cervical cancer [66], renal clear cell carcinoma [67] and tongue cancer [68] as an independent factor. Our meta-analysis showed that FHIT expression was positively linked to the favorable prognosis of the patients with gastric cancer. In contrast, our bioinformatics data indicated that FHIT mRNA expression was negatively associated with overall and progression-free survival rates of the patient with gastric cancer, even stratified by clinicopathological features. The paradoxical results are not strange since mRNA levels do not usually predict the corresponding protein levels because it takes a long distance from mRNA to functional protein by translation and posttranslational modification.

In conclusion, FHIT expression underwent a down-regulation during gastric carcinogenesis as a late event. It was negatively correlated with depth of invasion, lymph node metastasis, distant metastasis, TNM staging and dedifferentiation of gastric cancer. FHIT expression might be employed as a good potential marker for favorable prognosis of gastric cancer patients, while it was the converse for its mRNA. The limitations of this study include the potential publication bias from positive results in articles and the survival data extraction from survival curves.

## MATERIALS AND METHODS

### Identification of eligible studies and data extraction

We performed a publication search using PubMed, Web of Science, BIOSIS, SciFinder and CNKI updated on March 14, 2017. The following search terms were used: (FHIT OR fragile histine triad) AND (gastric OR stomach) AND (cancer OR carcinoma OR adenocarcinoma). Searching was done without restriction on language or publication years. Inclusion criteria for studies: (1)articles to observe the alteration in FHIT expression in gastric cancer by immunohistochemistry; (2) papers to compare FHIT expression with pathobiological behaviors and prognosis of gastric cancer by immunohistochemistry. Exclusion criteria included: (1) abstract, comment, review and meeting; (2) duplication of the previous publications; (3) Western blot, RT-PCR, cDNA microarray, or transcriptomic sequencing for FHIT expression; (4) lack of sufficient information.

### Data extraction

Based on the inclusion criteria, two reviewers (HC Zheng and LL Liu) independently extracted information from all eligible publications. The following information were included in each study: name of first author, year of publication, country, ethnicity, cancer types, source of control, antibody company, numbers of cases and controls, expression alteration, correlation with aggressive features, and follow-up times. Regarding survival analysis, we used Engauge Digitizer software to extract data from Kaplan-Meier curves and calculated the Hazard ratios (HR) and their corresponding 95% confidence intervals (CI). Any disagreement was resolved through discussion until the two reviewers reached a consensus.

### Quality score assessment

Two reviewers (HC Zheng and LL Liu) independently assessed the quality of the included studies according to Newcastle Ottawa Scale (NOS) (http://www.ohri.ca/programs/clinical_epidemiology/oxford.htm). The scale consists of three components related to sample selection, comparability and ascertainment of outcome.

### Bioinformatics analysis

The individual gene expression level of FHIT was analyzed using Oncomine (www.oncomine.org), a cancer microarray database and web-based data mining platform for a new discovery from genome-wide expression analyses. We compared the differences in FHIT mRNA level between gastric normal tissue and cancer. All data were log-transformed, median centered per array, and standard deviation normalized to one per array. The expression data (RNA-seqV2) and clinicopathological data of 392 gastric cancer patients were downloaded from the Cancer Genome Atlas (TCGA) database by TCGA-assembler in R software. We integrated the raw data, analyzed FHIT expression in gastric cancer, and compared it with clinicopathological and prognostic data of the patients with gastric cancer. Additionally, the prognostic significance of FHIT mRNA was also analyzed using Kaplan-Meier plotter (http://kmplot.com).

### Statistics analysis

HWE was evaluated using Chi-square test in control groups of each study. Strength of association between FHIT expression and cancer risk was assessed by odds ratios with 95% confidence intervals. Statistical significance of the pooled OR was determined by *Z* test. If there was no significant heterogeneity, the fixed effect model (Mantel-Haenszel method) would be employed. Otherwise, the random effect model (DerSimonian and Laird method) would be used excluding prognostic analysis. Heterogeneity effect was then quantified by I^2^ test, which was subdivided into low, moderate and high degrees of heterogeneity according to the cut-off values of 25%, 50% and 75% respectively. Publication bias was evaluated by funnel plot and quantified by Begg's test and Egger's test to assess funnel plot asymmetry. Meta-analyses were performed with Revman software 5.3 and data from TCGA database was dealt with SPSS 10.0 software using student *t* test. Two-sided *p* < 0.05 was considered as statistically significant.
